# A rare coding allele in *IFIH1* is protective for psoriatic arthritis

**DOI:** 10.1136/annrheumdis-2016-210592

**Published:** 2017-06-09

**Authors:** Ashley Budu-Aggrey, John Bowes, Philip E Stuart, Matthew Zawistowski, Lam C Tsoi, Rajan Nair, Deepak Rohit Jadon, Neil McHugh, Eleanor Korendowych, James T Elder, Anne Barton, Soumya Raychaudhuri

**Affiliations:** 1 Arthritis Research UK Centre for Genetics and Genomics, Centre for Musculoskeletal Research, University of Manchester, Manchester, UK; 2 NIHR Manchester Musculoskeletal Biomedical Research Unit, Central Manchester Foundation Trust and University of Manchester, Manchester Academy of Health Sciences, Manchester, UK; 3 Department of Dermatology, University of Michigan, Ann Arbor, Michigan, USA; 4 Department of Biostatistics, Center for Statistical Genetics, University of Michigan, Ann Arbor, Michigan, USA; 5 Royal National Hospital for Rheumatic Diseases, University of Bath, Bath, UK; 6 Department of Rheumatology, Cambridge University Hospitals NHSFT, Cambridge, UK; 7 Department of Pharmacy and Parmacology, University of Bath, Bath, UK; 8 Ann Arbor Veterans Affairs Hospital, Ann Arbor, Michigan, USA; 9 Department of Medicine, Brigham and Women’s Hospital and Harvard Medical School, Boston, Massachusetts, USA; 10 Program in Medical and Population Genetics, Broad Institute of MIT and Harvard, Cambridge, Massachusetts, USA

**Keywords:** *IFIH1*, association study, rare coding allele

## Abstract

**Objectives:**

Psoriatic arthritis (PsA) is an inflammatory arthritis associated with psoriasis. While many common risk alleles have been reported for association with PsA as well as psoriasis, few rare coding alleles have yet been identified.

**Methods:**

To identify rare coding variation associated with PsA risk or protection, we genotyped 41 267 variants with the exome chip and investigated association within an initial cohort of 1980 PsA cases and 5913 controls. Genotype data for an independent cohort of 2234 PsA cases and 5708 controls was also made available, allowing for a meta-analysis to be performed with the discovery dataset.

**Results:**

We identified an association with the rare variant rs35667974 (p=2.39x10^−6^, OR=0.47), encoding an Ile923Val amino acid change in the *IFIH1* gene protein product. The association was reproduced in our independent cohort, which reached a high level of significance on meta-analysis with the discovery and replication datasets (p=4.67x10^−10^). We identified a strong association with *IFIH1* when performing multiple-variant analysis (p=6.77x10^−6^), and found evidence of independent effects between the rare allele and the common PsA variant at the same locus.

**Conclusion:**

For the first time, we report a rare coding allele in *IFIH1* to be protective for PsA. This rare allele has also been identified to have the same direction of effect on type I diabetes and psoriasis. While this association further supports existing evidence for *IFIH1* as a causal gene for PsA, mechanistic studies will need to be pursued to confirm that *IFIH1* is indeed causal.

## Introduction

In approximately 6%–42% of cases psoriasis is associated with an inflammatory arthritis,^1^ psoriatic arthritis (PsA), which includes joint inflammation, involving primarily the distal interphalangeal (DIP) joints, entheses and sacroiliac joints. As patients typically test negative for rheumatoid factor, PsA is considered one of the spondyloarthropathies.^1^ Patients with PsA have a worse quality of life than patients with psoriatic skin disease alone.^2^ PsA may demonstrate a greater genetic component than psoriasis; a recent family study among first-degree relatives estimated a sibling recurrence risk (λ_s_) between 30 and 40, greater than that of psoriasis, where the recurrence risk is between 4 and 10.^3 4^

Genome-wide association studies have identified common risk alleles that are associated with both psoriasis and PsA.^5–10^ Familial studies^11^ and case-control studies^12^ have reported rare missense alleles in *CARD14* that confer strong risk of psoriasis, but to date no rare coding alleles have been identified for PsA risk. As rare coding variants are more likely to result in an interpretable functional effect, they might more easily provide insight into the mechanism of disease than known common variants associated with psoriasis and PsA. If rare alleles are protective, have a strong effect and result in loss of gene function, they could be flagging promising drug targets.^13^ For example, the Y142X, C679X and R46L alleles in *PCSK9* identified this gene as a promising drug target for lipid modification.^14 15^

To identify rare alleles that are associated with PsA (MAF<0.05), we queried the exome by analysing 1980 PsA cases and 5913 healthy controls that were genotyped using the Illumina HumanExome chip and HumanCoreExome chip. In doing so, we report an association with a rare coding variant at the *IFIH1* locus.

## Methods

### Study sample description

We recruited a total of 2384 patients with PsA and 5946 healthy individuals in this study following approval of the research ethics committee (MRES 99/8/84), as described previously.^16^ All participants were the UK Caucasians and had provided written informed consent.

### Genotyping

We genotyped DNA samples using the Infinium HumanExome-12 BeadChip (V.1-0) (Illumina) and the Infinium HumanCoreExome-24 BeadChip (V.1-0) (Illumina) (see online supplementary table 1), where 691 cases and all control samples were genotyped with the HumanExome chip, and the remaining 1693 cases were genotyped with the HumanCoreExome chip within the Arthritis Research UK Centre for Genetics and Genomics at The University of Manchester.

10.1136/annrheumdis-2016-210592.supp1 Supplementary file

### Genotype calling and quality control

We performed genotype calling and clustering with the GenomeStudio Data Analysis software (Genotyping Module V.1.8.4). We carried out initial genotype clustering to identify and exclude samples with a genotyping call rate <98%. We then performed automated reclustering of the remaining samples to obtain more accurate genotype clusters. As we were concerned that rare variants may be incorrectly clustered, we conducted an extensive manual review of clusters. We reviewed variants that were filtered based on cluster separation (<0.4), signal intensity (<1.0) and allele frequency, where we identified variants that had failed genotyping, and those that had been incorrectly called as ‘missing’.

### Statistical quality control

We identified duplicated samples and related individuals using identity-by-descent with PLINK software (see online supplementary table 2). We also performed principal component analysis with the EIGENSOFT software package (V.6.0.1), to identify and exclude samples with divergent ancestry (see online supplementary figure 1, supplementary table 2). Principal components were calculated with a set of linkage disequilibrium (LD)-pruned single nucleotide polymorphisms (SNPs) (n=13 444). We excluded monomorphic, Y chromosome, pseudoautosomal, mitochondrial and variants within the human leukocyte antigen (HLA) region, as well as variants with a call rate <100% in order to ensure high quality of the data. We also excluded SNPs found to significantly deviate from Hardy-Weinberg equilibrium (<1.0×10^−6^) among the control samples.

To prevent any bias due to differences in genotyping between the two platforms used in this study, we excluded all variants with evidence of case allele frequency differences between the two platforms (p<0.05) (see online supplementary figure 2). We also excluded variants with an allele count of 0 among either of the case cohorts or the control samples. Furthermore, we excluded rare variants showing evidence of genotyping bias by assessing the allele counts of cases and controls for each genotyping platform used.

### Replication dataset

We used an independent cohort of 2234 PsA cases and 5708 controls to replicate our findings. Briefly, people of European Caucasian ancestry were collected in North America and Sweden, and were genotyped using the Affymetrix Axiom Biobank Plus Genotyping Array. More details of this cohort, as well as quality control procedures applied to the genotype data, have been described previously.^17^

### Association testing

We performed single-point analysis using the Fisher's exact test in PLINK with all SNPs passing quality control, and separately for rare variants. We calculated genomic inflation for all variants, and separately for rare and common variants alone, where we created a null SNP set (n=13 106) with the common variants and excluded previously reported psoriasis loci (see online supplementary figures 3–5). We performed multiple-variant testing with the rare variants using SKAT analysis in R. We used the Firth logistic regression test (with four principal components as covariates) for association analysis of the replication cohort. In our replication cohort, principal components had been calculated with a set of LD-pruned SNPs, where known psoriasis loci had been excluded (n=11 005). We performed a meta-analysis with the discovery and independent cohort data assuming an inverse-variance fixed-effects model. The choice of methods to perform the single-variant analysis was based on their suitability for analysing rare variants.

### *IFIH1* conditional analysis and conditional haplotype analysis

At the *IFIH1* locus, we performed conditional logistic regression in PLINK to test for independence between the rare (Ile923Val) and common (rs984971) variants using PsA Immunochip genotype data (1962 cases, 8923 controls). We also used this dataset to construct haplotypes in PLINK to test for association with PsA, where we calculated OR and 95% CIs. We performed conditional haplotype analysis based on a likelihood ratio, to test for an effect of Ile923Val independent from that of rs984971.

## Results

After quality control, we examined genotype data for 41 267 non-HLA variants in 1980 cases and 5913 controls (see online supplementary table 3). We excluded more case samples compared with controls due to the overlap of case samples that were genotyped on both platforms. We carried out single-point analysis using the Fisher's exact test to identify associations with PsA. Unsurprisingly, we observed evidence of association in our cohort at known PsA alleles (see online supplementary table 4), including the rs33980500 SNP at *TRAF3IP2* (p=3.9×10^−18^). After removing these known associations, we observed only modest genomic inflation (λ=1.06, λ_1000,1000_=1.02), suggesting little evidence of population stratification (see online supplementary figure 4).

We conducted a single-point analysis for a total of 27 066 rare variants alone (MAF<0.05, λ=0.79) (see online supplementary figure 5). The strongest association we found was with the SNP rs35667974 (p=2.39×10^−6^, OR=0.47) (see online supplementary figures 6 and 7, supplementary table 5), mapping to *IFIH1*; this variant causes a missense mutation, resulting in an amino acid substitution of valine for isoleucine (Ile923Val). Of the 18 variants taken forward for replication (p<1.0×10^−3^), we only found the Ile923Val association to replicate within our independent cohort (p=3.5×10^−5^, OR=0.49), based on a Bonferroni-corrected p value threshold of 2.78×10^–3^ (=0.05/18, see online supplementary tables 6 and 7). On meta-analysis with the discovery and replication datasets, we found the association with the Ile923Val allele to exceed genome-wide significance (p=4.78×10^-10^). We performed multiple-variant analysis to assess allele counts among genes and obtained further evidence to support the association of *IFIH1* with PsA (p=6.77×10^−6^, Bonferroni-corrected p value threshold=5.60×10^−6^, based on the number of genes tested) (see online supplementary table 8).

### Independent effects at the *IFIH1* locus

**Figure 1:**
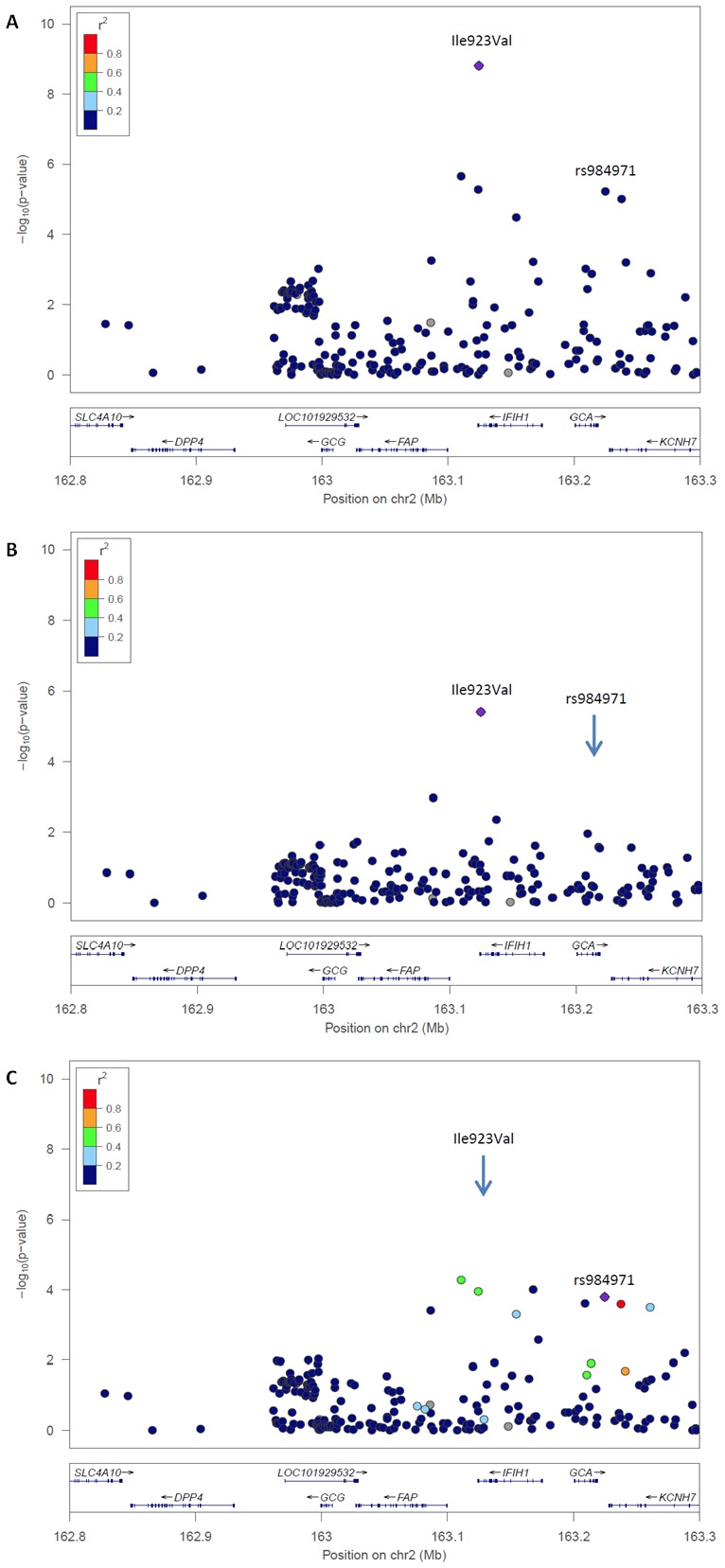
Conditional logistic regression within the *IFIH1* region using psoriatic arthritis (PsA) Immunochip genotype data (Bonferroni-corrected p<0.025). (A) Association of the rare (Ile923Val) and common variant (rs984971) with PsA (Fisher’s exact test). (B) The rare variant remains associated when rs984971 is conditioned on. (C) The common variant remains associated when the Ile923Val variant is conditioned on. ↓ Position of the SNP that has been conditioned on.

**Figure 2:**
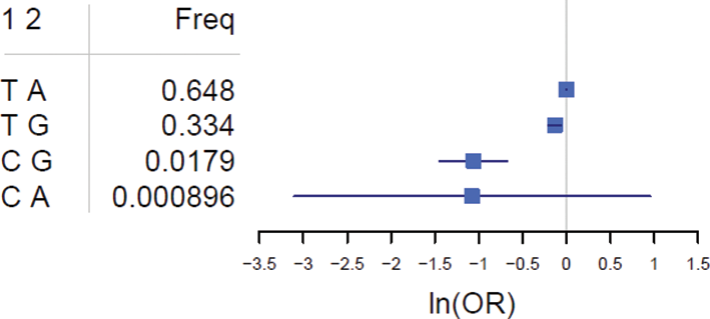
Frequencies and psoriatic arthritis association OR of the haplotypes for Ile923Val and rs984971. OR and their 95% CIs are shown relative to the most frequent haplotype on a natural log scale (ln). 1; Ile923Val, 2; rs984971, Freq; frequency.

A common variant at *IFIH1* has been previously reported as associated with PsA in our recent Immunochip study.^9^ When performing single-point analysis with the Immunochip dataset, we found evidence for an independent signal within the locus. In that study, we observed that Ile923Val obtained nominal significance after conditional analysis (figure 1). We also observed this association to be stronger than that previously reported for the common variant at the same locus, rs984971 (p=3.62×10^−6^). When we performed conditional logistic regression, the Ile923Val variant remained associated with PsA (p<0.05) while conditioning on rs984971 (p_cond_=4.0×10^−6^) (figure 1). Likewise, the SNP rs984971 remained associated with PsA when we conditioned on the Ile923Val variant (p_cond_=1.6×10^−4^) (figure 1). This gives evidence of the independent effects of the two variants. We performed conditional haplotype analysis using haplotypes constructed with both variants (figure 2). Here, we found that the derived haplotype containing Ile923Val (CG) has a significantly greater protective effect compared with that containing the derived allele at rs984971 (TA), relative to the ancestral haplotype (TG) (p=6.36×10^−11^ vs p_cond_=2.95×10^−7^). This confirms that the association with the rare *IFIH1* variant is not driven by the common rs984971 variant.

## Discussion

We have identified a PsA association with the rare coding allele Ile923Val within the *IFIH1* gene. This rare allele was found to be protective for PsA, and also replicated within an independent cohort. We found the association signal to reach genome-wide significance during meta-analysis, and confirmed the association with *IFIH1* when performing multiple-variant analysis. The Ile923Val allele has been previously reported to be protective for type 1 diabetes^18^ and psoriasis.^19^ Associations within the *IFIH1* locus have also been reported to increase PsA risk.^10 20^ However, until now no association with the Ile923Val allele has been reported to have a protective effect on PsA alone. We found the Ile923Val variant to be independent of the common SNP previously identified at the *IFIH1* locus (rs984971). This SNP is highly correlated (r^2^=0.64) with an additional common *IFIH1* variant (rs1990760) that has been recently reported for PsA.^10^ We also found the Ile923Val variant to have an independent effect from this variant (P_cond_=4.39×10^−6^). Furthermore, we also found the Ile923Val variant to be independent of the common rs3747517 *IFIH1* variant previously reported for PsA (P_cond_=7.73×10^−7^).^10^ These findings highlight the relevance of *IFIH1* in immune-related disorders, especially given the recent association reported for the rare *IFIH1* allele with ankylosing spondylitis, Crohn's disease, psoriasis, primary sclerosing cholangitis and ulcerative colitis.^21^ The frequency of the Ile923Val variant in our control population (0.021) is slightly higher than observed in European populations in the 1000 genomes project and the exome sequencing project (0.01–0.018).^22 23^ This should be taken into consideration when interpreting the results of this study.

As the rare allele identified lies within a coding region, this provides strong evidence to suggest that *IFIH1* is a causal gene for PsA. As discussed by Plenge *et al*,^13^ a rare and independent variant in a causal gene for disease could provide a potential therapeutic target for treatment. However, linking human genetics with therapeutic targets requires the biological function of the causal gene and variant to be known.

*IFIH1* encodes a cytoplasmic RNA helicase that recognises viral RNA and mediates an immune response on viral infection.^18^ The protective effect of the rare *IFIH1* allele observed with PsA suggests that the variant results in a loss of function phenotype, where the production or activity of *IFIH1* is decreased. Increased expression levels of *IFIH1* have been observed in psoriasis skin lesions compared with healthy skin,^10^ suggesting that pharmacological inhibition of this gene could be effective in treating psoriatic disease. However, when investigating the effect of Ile923Val in knockout mice, mutants showed no change in their double-stranded RNA binding activity compared with the wild-type.^24^ This indicates the requirement for further investigation into the biological effect before any conclusions can be drawn from our current study regarding the impact of this allele on disease or the potential for treatment of PsA. Mechanistic studies will also be required to confirm that *IFIH1* is causal for PsA.

In conclusion, we report for the first time an association with a rare coding allele in *IFIH1* that is protective for PsA, and independent of associations that have previously been reported within this locus.
