# Supplementary material for: A rare coding allele in IFIH1 is protective for psoriatic arthritis
Source: Ann Rheum Dis. 2017 Jun 9;76(7):1321–4. doi: 10.1136/annrheumdis-2016-210592 (PMC5530346; doi:10.1136/annrheumdis-2016-210592)
Supplement: Supplementary file [file annrheumdis-2016-210592supp001.pdf]

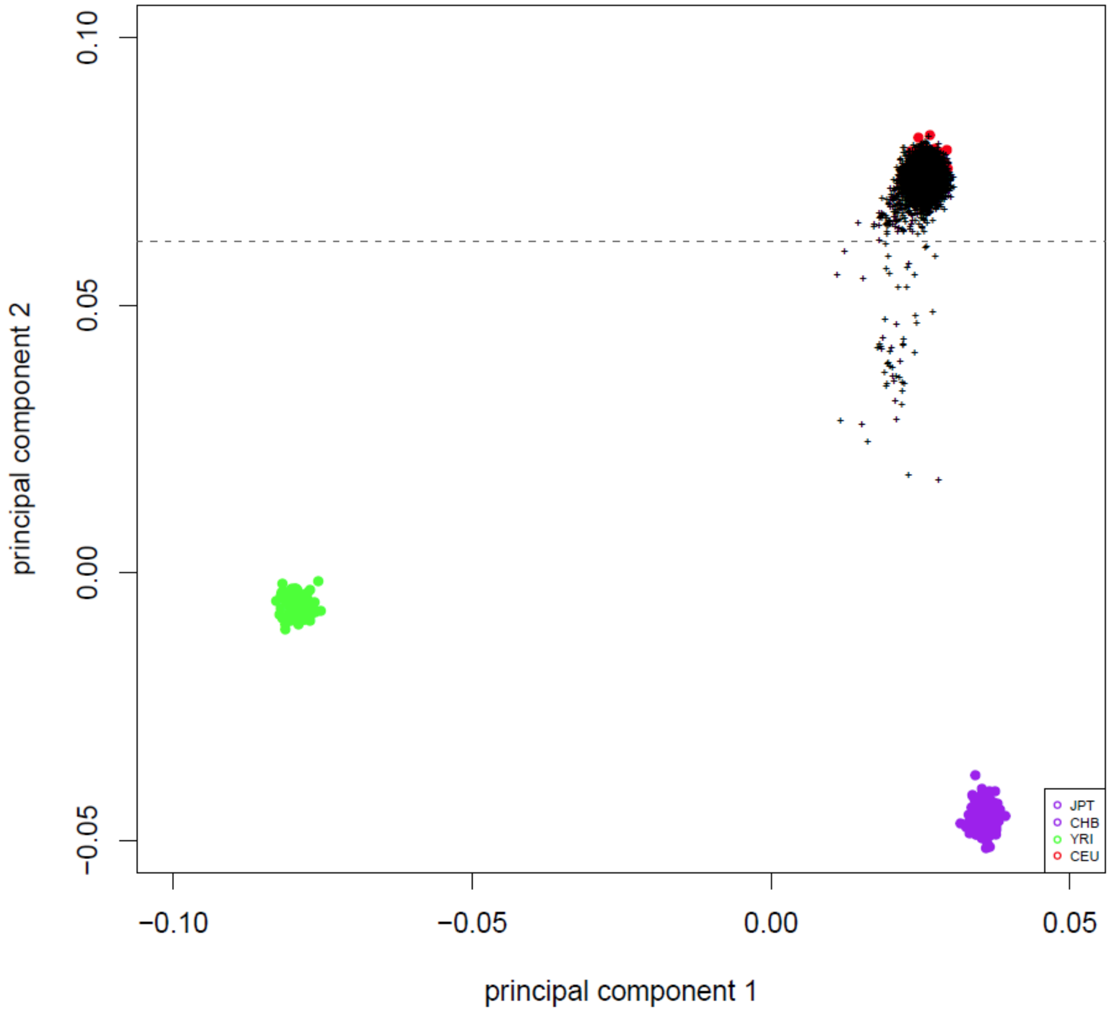

Supplementary Figure 1: Principal component analysis of study samples against HapMap population reference panels of Asian (JPT and CHB), African (YRI) and European (CEU) populations. Samples below horizontal dashed line were excluded (second principal component score < 0.062)

+ indicates discovery cohort samples

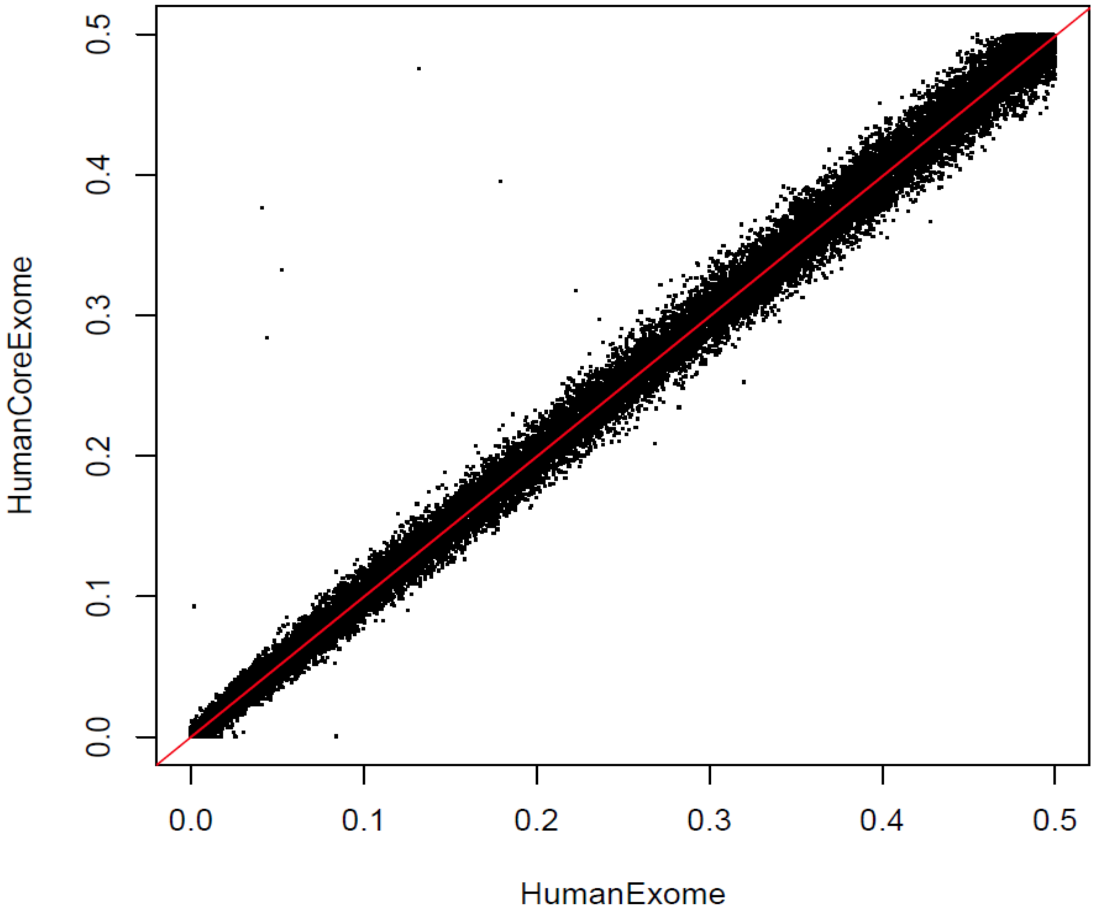

Supplementary Figure 2: Scatter plot of correlation of allele frequencies between cases genotyped with the HumanExome chip and HumanCoreExome chip. r = 0.99

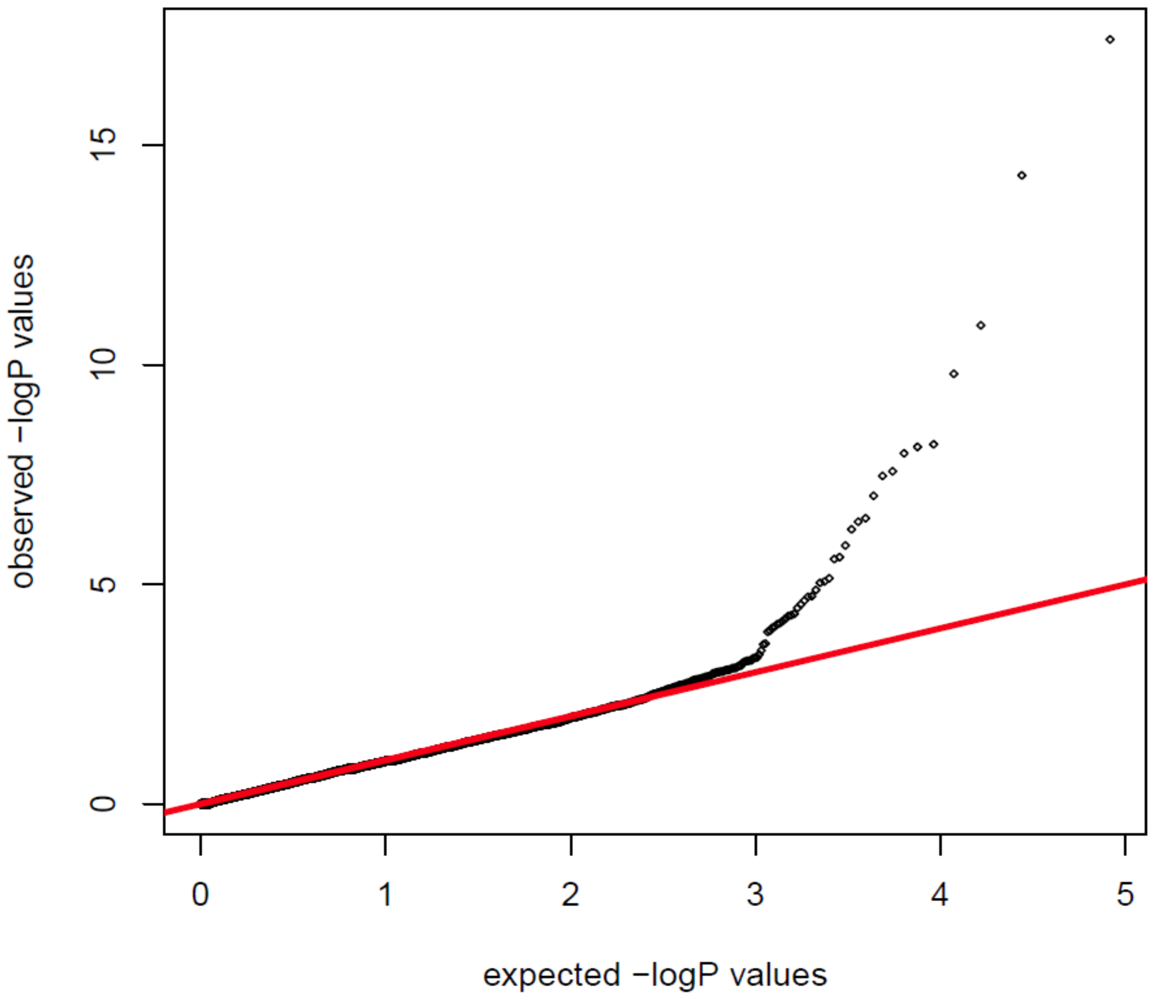

Supplementary Figure 3: Quantile-quantile (Q-Q) plot of Fisher's Exact *P*-values for common and rare alleles. Genomic inflation factor (λ) = 0.9

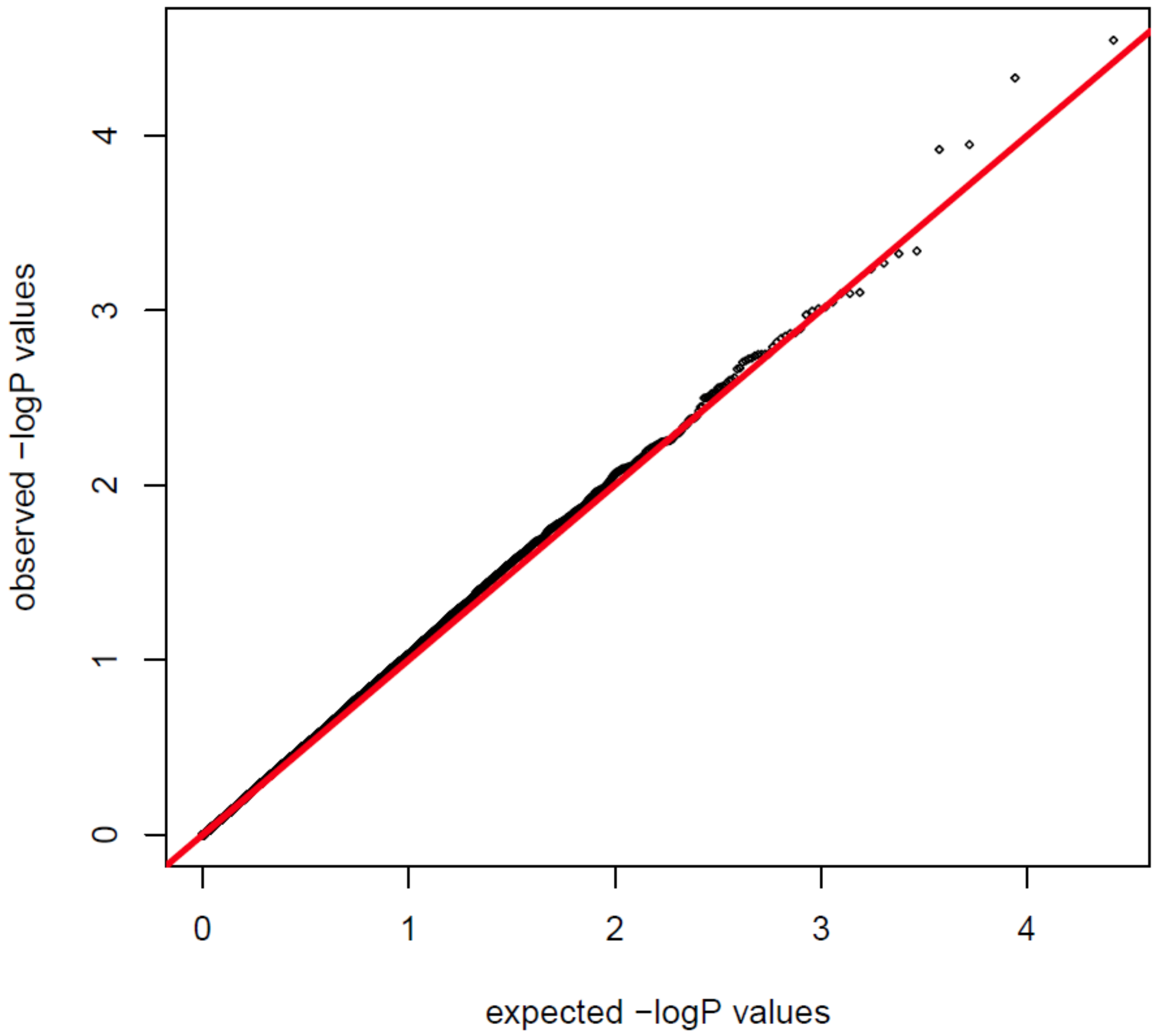

Supplementary Figure 4: Quantile-quantile (Q-Q) plot of Fisher's Exact *P*-values for common alleles with variants in regions of known association removed (n=13,105, MAF>0.05). Genomic inflation factor (λ) = 1.06

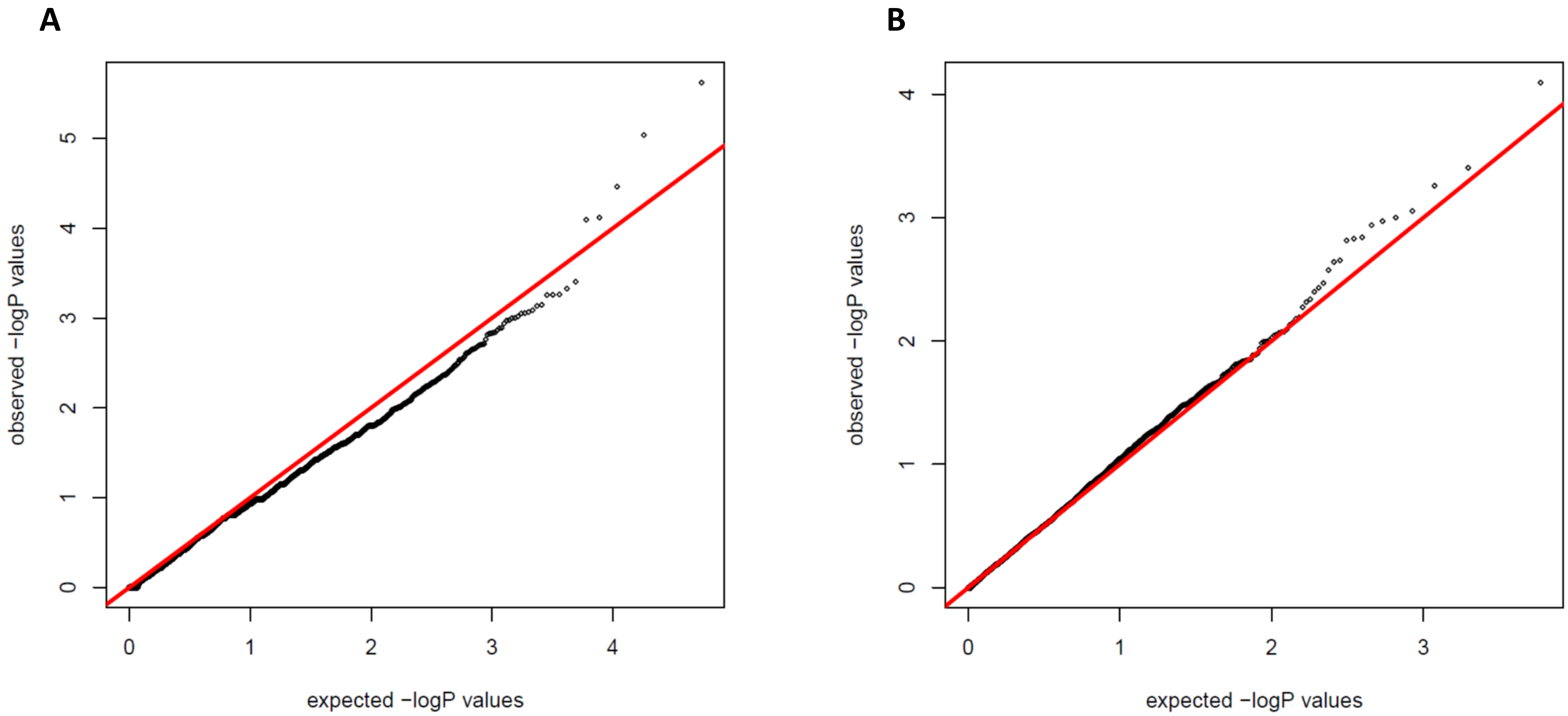

Supplementary Figure 5: Quantile-quantile (Q-Q) plot of Fisher's Exact *P*-values for rare alleles [a].
(A) MAF < 0.05. Genomic inflation factor (λ) = 0.79.
(B) MAF 0.02-0.05 (n = 2,960). Genomic inflation factor (λ) = 1.01.

[a] Deflation observed in the Q-Q plot is due to the large proportion of extremely rare variants (MAF < 0.02)

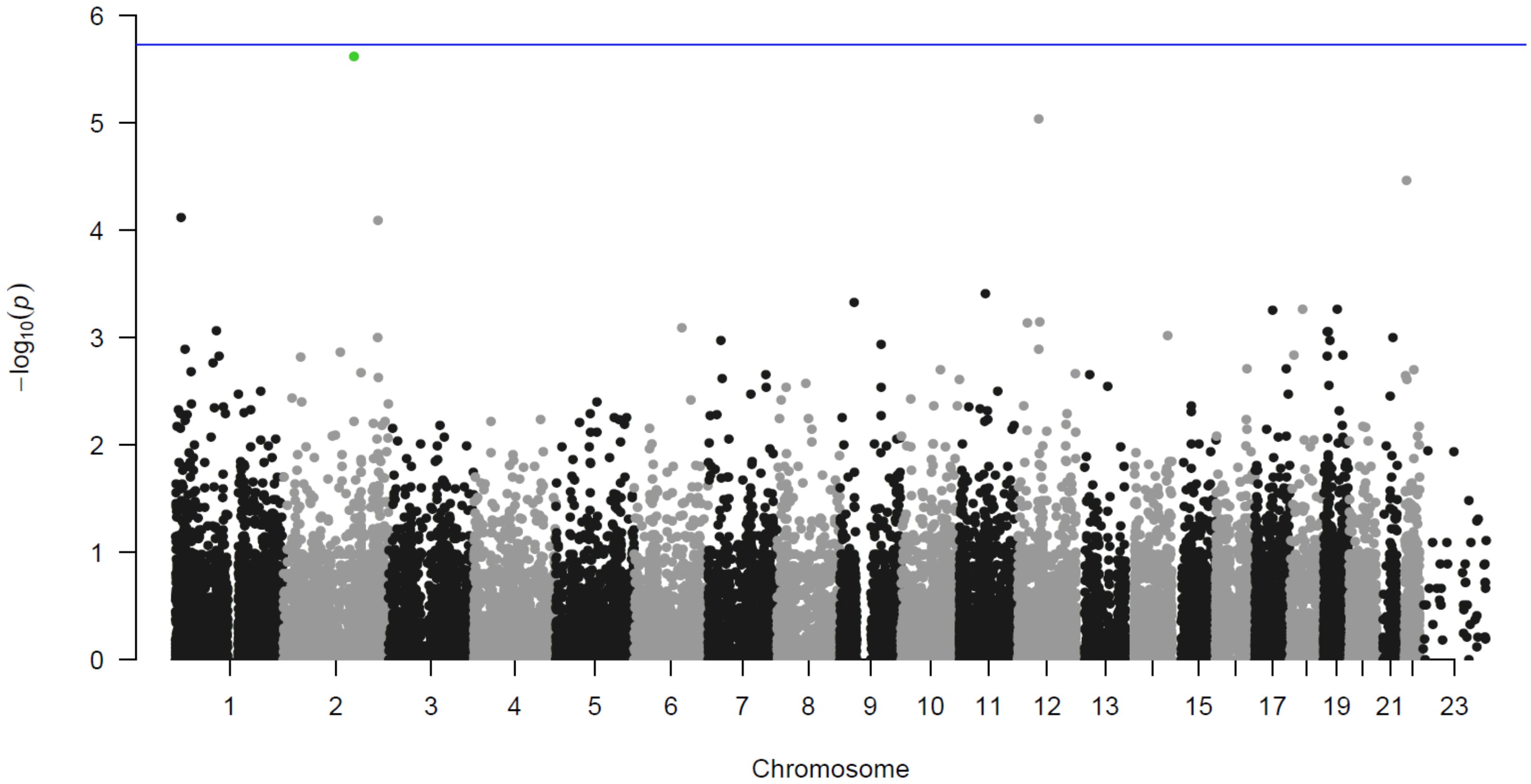

Supplementary Figure 6: Manhattan plot of results of single-point analysis of rare (27,066) variants. Chromosomal location is plotted on the x-axis and the -log10 of the observed *P* value is plotted on the y-axis. The most strongly associated variant (Ile923Val) is highlighted in green. The blue line indicates the corrected significance threshold based on the number of markers tested ($P_{corr}=1.8\times10^{-6}$).

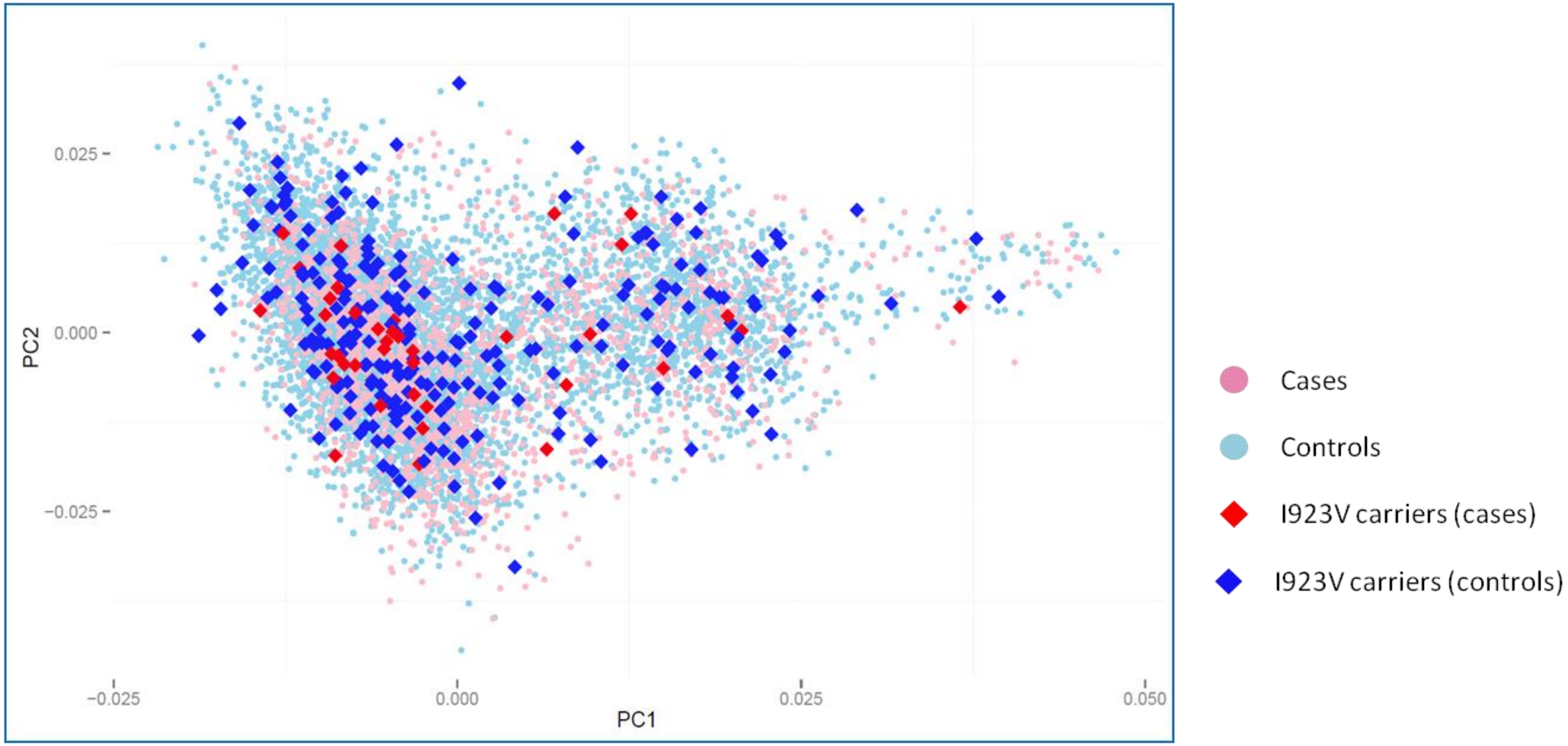

Supplementary Figure 7: Here, we plot genotyped individuals as a function of their principal components based on genotype data to identify any evidence of population stratification. We calculated principal components with a set of 13,444 LD pruned variants. We have plotted cases and controls, as well as those who are heterozygous for the *IFIH1* Ile923Val variant, with respect to the first two components.

Supplementary table 1: Summary of genes and markers present on the HumanExome chip and the HumanCoreExome chip

**HumanExome genes (exons)**	**18,245 (212,850)**
**HumanExome markers**	247,870
**HumanCoreExome genes (exons)**	19,289 (228,271)
**HumanCoreExome markers**	547,644
**Unique HumanExome markers**	5,152
**Unique HumanCoreExome markers**	304,926
**Overlapping markers**	242,718

Supplementary table 2: Summary of exclusions made during sample quality control within the discovery cohort. QC; quality control

Sample QC	Samples remaining	Samples excluded
**START**	2,384 cases<br>5,946 controls	
Related individuals	2,324 cases<br>5,917 controls	60 cases<br>29 controls
Duplicated samples	2,030 cases<br>5,917 controls	294 cases
Divergent ancestry	1,980 cases<br>5,913 controls	50 cases<br>4 controls
**FINISH**	**1,980 cases<br>5,913 controls**	

Supplementary table 3: Summary of exclusions made during SNP quality control within the discovery cohort. QC; quality control, MAF; minor allele frequency, MT; mitochondria, HWE; Hardy-Weinberg equilibrium, MHC; major histocompatibility complex

SNP QC	SNPs remaining	SNPs Excluded
**START**	242, 718	
Different MAFs between cases	225,427	17,291
Chromosome XY/ Y/MT	224,848	579
Monomorphic	121,565	103,283
Call rate (<1.0)	48,559	73,006
HWE (P<1x10^{-6})	48,081	478
Allele count <1.0	42,928	5,153
Minus MHC region	41,554	1,374
Total excluded		201,164
**FINISH (all variants)**	**41,554**	
**FINISH (rare and low-frequency variants)**	**27,353**	14,201
**FINISH (Minus differences in allele counts**	**27,066**	287

Supplementary table 4: Summary statistics of common variants identified at previously reported PsA and psoriasis GWAS loci (Fisher's Exact test). [a]allele labels are given in relation to the forward strand, Chr; chromosome, MAF; minor allele frequency, OR; odds ratio (in reference to the minor allele)

SNP	Chr	Position (HG19)	Minor/major allele[a]	MAF (case)	MAF (control)	*P* value	Allelic OR	Gene	Amino acid change	Stuart SNP[1]	r^2 Stuart SNP[1]
rs33980500	6	111913262	T/C	0.11	0.07	$3.88\text{x}10^{-18}$	1.74 (1.54 - 1.97)	*TRAF3IP2-AS1*	D10N (Missense)	rs33980500	1.00
rs11209003	1	67601132	T/G	0.35	0.29	$1.26\text{x}10^{-11}$	1.31 (1.21 - 1.41)	*IL23R*	Intergenic	rs12044149	0.81
rs6871626	5	158826792	A/C	0.29	0.34	$1.03\text{x}10^{-8}$	0.80 (0.74 - 0.86)	*IL12B*	Intergenic	rs918520	0.11
rs2066807	12	56740682	G/C	0.04	0.07	$9.58\text{x}10^{-8}$	0.65 (0.55 - 0.76)	*STAT2*	M594I (Missense)	rs2066807	1.00
rs20541	5	131995964	A/G	0.14	0.18	$3.74\text{x}10^{-7}$	0.77 (0.70 - 0.86)	*IL13*	Q144R (Missense)	rs1295685	0.98
rs35667974	2	163124637	G/A	0.01	0.02	$2.39\text{x}10^{-6}$	0.47 (0.33 - 0.66)	*IFIH1*	I923V (Missense)	rs1990760	0.02
rs2304256	19	10475652	A/C	0.25	0.29	$2.65\text{x}10^{-6}$	0.82 (0.76 - 0.89)	*TYK2*	V362F (Missense)	rs34536443	0.08
rs10903122	1	25303576	A/G	0.46	0.50	$1.34\text{x}10^{-5}$	0.85 (0.79 - 0.92)	*RUNX3*	Intergenic	rs11249215	0.89
rs2235617	20	48554977	C/G	0.38	0.42	$1.85\text{x}10^{-5}$	0.85 (0.79 - 0.92)	*RNF114*	Silent	rs2235617	1.00
rs10865331	2	62551472	A/G	0.41	0.37	$2.33\text{x}10^{-5}$	1.17 (1.09 - 1.26)	*B3GNT2*	Intergenic	rs10865331	1.00
rs4795067	17	26106675	G/A	0.39	0.35	$5.03\text{x}10^{-5}$	1.17 (1.08 - 1.26)	*NOS2*	Intergenic	rs28998802	0.03
rs702873	2	61081542	A/G	0.39	0.42	$5.27\text{x}10^{-05}$	0.86 (0.80 - 0.92)	*FLJ16341*	Silent	rs34958906	0.99
rs610604	6	138199417	C/A	0.36	0.32	$9.69\text{x}10^{-5}$	1.16 (1.08 - 1.26)	*TNFAIP3*	Silent	rs610604	1.00
rs8016947	14	35832666	T/G	0.41	0.44	$9.61\text{x}10^{-4}$	0.88 (0.82 - 0.95)	*NFKBIA*	Intergenic	rs12883343	0.44

rs30187	5	96124330	T/C	0.36	0.34	1.24×10^{-3}	1.13 (1.05 - 1.22)	*ERAP1*	K528R (Missense)	rs30377	0.45
rs181359	22	21928641	T/C	0.22	0.19	1.25×10^{-3}	1.16 (1.06 - 1.27)	*UBE2L3*	Silent	rs5754387	0.97
rs8102380	19	10801185	G/A	0.32	0.34	3.98×10^{-3}	0.89 (0.83 - 0.96)	*ILF3*	Silent	rs873016	0.54
rs149000560	11	63974966	A/G	0.01	0.01	4.77×10^{-3}	1.66 (1.18 - 2.35)	*FERMT3*	G44R (Missense)	rs887314	0.01
rs12928822	16	11403893	T/C	0.17	0.19	6.13×10^{-3}	0.88 (0.80 - 0.96)	*PRM3, SOCS1*	Intergenic	rs413024	0.39
rs6908425	6	20728731	T/C	0.20	0.22	0.02	0.90 (0.82 - 0.98)	*CDKAL1*	Silent	rs4712528	0.97
rs11538189	1	24710405	G/A	0.12	0.13	0.03	0.89 (0.80 - 0.99)	*C1orf201*	M93T (Missense)	rs7540214	0.02
rs10462021	1	7897133	G/A	0.21	0.19	0.04	1.10 (1.00 - 1.20)	*PER3*	H1149R (Missense)	rs12041676	0.01
rs2075641	10	75562108	A/G	0.12	0.13	0.04	0.89 (0.80 - 1.00)	*NDST2*	Silent	rs2675669	0.15

[1] Stuart PE, Nair RP, Tsoi LC et al. Genome-wide Association Analysis of Psoriatic Arthritis and Cutaneous Psoriasis Reveals Differences in Their Genetic Architecture. *Am J Hum Genet* 2015;**97**:816-36.

Supplementary table 5: Summary statistics of the strongest associations identified ($P < 1.0x10^{-4}$) when analysing rare variants (Fisher's Exact test). [a]allele labels are given in relation to the forward strand, Chr; chromosome, MAF; minor allele frequency, OR; odds ratio (in reference to the minor allele), EC; HumanExome chip, HCE; HumanCoreExome chip

SNP	Chr	Position (HG19)	Minor/ major allele[a]	MAF (case)	MAF (control)	*P* value	Allelic OR	Gene	Allele count: 686 EC cases	Allele count: 1294 HCE cases	Allele count: 5913 controls	Amino acid change
rs35667974	2	163124637	C/T	0.010	0.021	$2.39x10^{-6}$	0.47 (0.33 - 0.66)	*IFIH1*	16	23	246	I923V (Missense)
rs148755202	12	48191247	T/C	0.010	0.020	$9.17x10^{-6}$	0.48 (0.34 - 0.68)	*HDAC7*	10	29	238	R166H (Missense)
rs138601828	22	21800049	A/G	0.007	0.002	$3.44x10^{-5}$	3.24 (1.88 - 5.59)	*HIC2*	7	20	25	V289I (Missense)
rs41303871	1	12836101	A/C	0.016	0.008	$7.61x10^{-5}$	1.94 (1.41 - 2.66)	*PRAMEF12*	22	41	98	L235I (Missense)
rs41272679	2	218762616	T/C	0.051	0.036	$8.05x10^{-5}$	1.43 (1.20 - 1.69)	*TNS1*	65	137	430	V25I (Missense)

Supplementary table 6: Summary statistics for the association at rs35667974 within the discovery and replication cohorts. [a]allele labels are given in relation to the forward strand, Chr; chromosome, MAF; minor allele frequency, OR; odds ratio (in reference to the minor allele)

						Genotype counts (CC/CT/TT)	
**Dataset**	**Minor/major allele[a]**	**MAF (case)**	**MAF (control)**	***P* value**	**Allelic OR**	**Cases**	**Controls**
Discovery	C/T	0.010	0.021	$2.39x10^{-6}$	0.47 (0.33 - 0.66)	0/39/1941	2/242/5669
Replication	C/T	0.008	0.018	$3.50x10^{-5}$	0.49 (0.34 - 0.70)	0/36/2198	0/203/5505

Supplementary table 7: Summary statistics for variants investigated within the replication cohort. [a]allele labels are given in relation to the forward strand, Chr; chromosome, MAF; minor allele frequency, OR; odds ratio (in reference to the minor allele), *variant not available for analysis

SNP	Chr	Position (HG19)	Minor/major allele[a]	MAF (case)	MAF (control)	*P* value	Allelic OR	$P_{Discovery}$	Gene	Amino acid change
rs35667974	2	163124637	C/T	0.008	0.018	$3.50x10^{-5}$	0.49 (0.34 - 0.70)	$2.39x10^{-6}$	*IFIH1*	I923V (Missense)
rs35018800	19	10464843	A/G	0.004	0.008	0.04	0.61 (0.36 – 0.98)	$8.89x10^{-4}$	*TYK2*	A928V (Missense)
rs61752945	9	32481339	T/C	0.008	0.010	0.12	0.75 (0.51 – 1.08)	$4.71x10^{-4}$	*DDX58*	R546Q (Missense)
rs35508987	17	40997461	A/G	0.012	0.009	0.13	1.30 (0.92 – 1.82)	$5.55x10^{-4}$	*AOC2*	R273Q (Missense)
rs11575194	2	217543728	A/G	0.047	0.044	0.26	1.10 (0.93 – 1.30)	$9.98x10^{-4}$	*IGFBP5*	R138W (Missense)
rs41303871	1	12836101	A/C	0.010	0.008	0.28	1.23 (0.84 – 1.77)	$7.61x10^{-5}$	*PRAMEF12*	L235I (Missense)
rs146045772	19	31040226	G/C	0.006	0.005	0.30	1.28 (0.80 – 2.02)	$5.46x10^{-4}$	*ZNF536*	(H1234D) Missense
rs41288586	6	110636603	T/C	0.004	0.006	0.50	0.84 (0.49 – 1.38)	$8.18x10^{-4}$	*METTL24*	(D167N) Missense
rs148755202	12	48191247	T/C	0.015	0.017	0.52	0.91 (0.68 - 1.20)	$9.17x10^{-6}$	*HDAC7*	(R166H) Missense
rs2336573	19	8367709	T/C	0.037	0.034	0.56	1.06 (0.88 – 1.27)	$8.84x10^{-4}$	*CD320*	(G220R) Missense
rs41272679	2	218762616	T/C	0.036	0.040	0.59	0.95 (0.79 – 1.14)	$8.05x10^{-5}$	*TNS1*	(V25I) Missense
rs117474773	12	21689946	G/A	0.007	0.007	0.69	0.92 (0.60 – 1.37)	$7.34x10^{-4}$	*GYS2*	(F685S) Missense
rs138601828	22	21800049	A/G	0.006	0.005	0.74	0.92 (0.56 – 1.47)	$3.44x10^{-5}$	*HIC2*	(V289I) Missense

rs1800555	1	94463617	T/C	0.012	0.012	0.77	1.05 (0.75 – 1.45)	$8.58x10^{-4}$	*ABCA4*	(D2177N) Missense
rs12787462	11	59573980	T/C	0.027	0.027	0.80	1.03 (0.83 – 1.27)	$3.94x10^{-4}$	*MRPL16*	(R199Q) Missense
rs7236477*	18	28902322	G/A					$5.51x10^{-4}$	*DSG1*	Silent
rs191603809*	12	50187579	A/C					$7.13x10^{-4}$	*NCKAP5L*	(A1066S) Missense
rs147639735	14	96730141	T/C	0.000	0.000	NA	NA	$9.59x10^{-4}$	*BDKRB1*	(P41L) Missense

Supplementary table 8: Summary statistics of *IFIH1* variants from single-variant analysis (Fisher's Exact), that were included in the multiple-variant analysis. [a]allele labels are given in relation to the forward strand, Chr; chromosome, MAF; minor allele frequency, OR; odds ratio (in reference to the minor allele).

SNP	Chr	Position (HG19)	Minor/major allele[a]	MAF (case)	MAF (control)	*P* value	Allelic OR	Amino acid change
rs35667974	2	163124637	C/T	0.010	0.021	2.39×10^{-6}	0.47 (0.33 - 0.66)	I923V (Missense)
rs35732034	2	163124596	T/C	0.006	0.009	0.04	0.62 (0.39 - 0.97)	Silent
rs35744605	2	163134090	A/C	0.004	0.007	0.06	0.59 (0.34 - 1.01)	E627X (Nonsense)
rs79324540	2	163128828	T/C	0.001	0.002	0.82	0.79 (0.29 - 2.11)	E842K (Missense)
rs72650663	2	163133396	A/G	0.004	0.003	0.88	1.05 (0.57 - 1.92)	T702I (Missense)
